# Acute myocarditis and pulmonary edema due to scorpion sting

**DOI:** 10.21542/gcsp.2016.10

**Published:** 2016-03-31

**Authors:** Montaser Ismail, Nidal Asaad, Jassim Al Suwaidi, Maryam Al Kawari, Amar Salam

**Affiliations:** Cardiology & Cardiac Surgery Department, Hamad Medical Corporation, Doha, Qatar

**Keywords:** scorpion sting, myocarditis, cardiogenic shock

## Abstract

**Objective:** To present a case of a serious manifestation of scorpion sting, which was not reported before in Qatar, review the literature, and compare with previously reported similar cases.

**Case presentation and intervention:** A young male patient was admitted to CCU with a clinical picture of acute toxic myocarditis and cardiogenic shock with abnormal ECG and elevated cardiac markers after a scorpion sting to his right big toe. Thorough investigations, including echocardiography, cardiac MRI and right heart catheterization, supported the diagnosis. Coronary angiography was normal. Patient was managed conservatively with supportive measures, mechanical ventilation, IV fluids, inotropic agents, steroids, antibiotics and Prazocin. Over 9 days of hospital course, patient gradually improved, was successfully extubated, and was discharged in a stable condition.

**Conclusion:** Toxic myocarditis (with myocardial damage), pulmonary edema and cardiogenic shock are reported manifestations of scorpion venom intoxication.

## Introduction

Creatures that are capable of producing a poison in a gland and then delivering that toxin by stinging or biting are called venomous animals. Arthropods (scorpions, spiders, bees, and wasps) are examples.

Scorpion envenomation is potentially fatal in many regions worldwide. Children form the majority of victims. The sting effect depends on the size of the victim, the season, the age of the offender and the delivery dose of the scorpion^1,3^. Several clinical syndromes with variable hemodynamic patterns may dominate the clinical presentation^2,3^.

**Figure 1.**
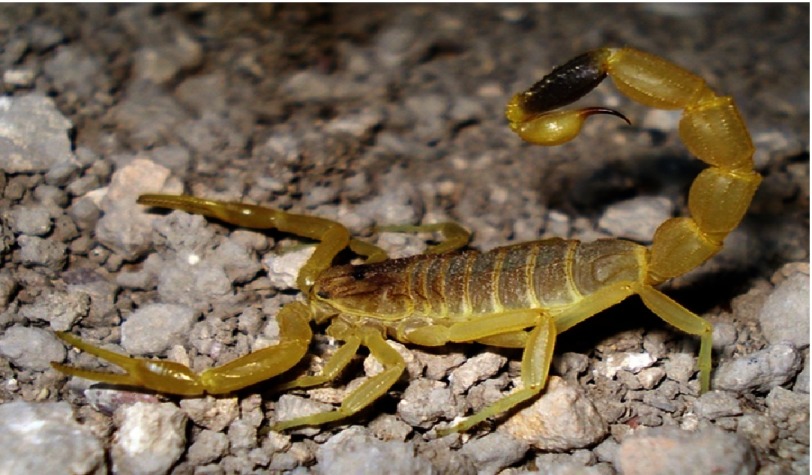
*Leiurus Quinquestriatus*: one of the comon scorpion species in the Middle East and Saudi Arabia.

There have been many case reports and reviews about scorpion sting-related myocardial dysfunction and hemodynamic disturbances, mainly in the endemic areas. The Middle East has a relatively high incidence of scorpion envenomation; however no similar cases were reported in Qatar previously. *Leiurus Quinquestriatus* (Figure 1), and *Androctonus crassicauda* are the most common scorpion offenders in the Gulf Area and Saudi Arabia^1^ (Table 1).

Here we present a case of myocardial damage and cardiogenic shock as a result of a scorpion envenomation.

## Case report

A 24-year-old Nepalese male patient who worked as a shepherd in a suburban farm in Doha, Qatar, and with no significant past medical history, presented to the emergency room with a sudden onset of nausea, vomitting, dizziness and profuse sweating around 30 minutes after a scorpion sting to the right big toe. The patient described the scorpion as “yellowish-white color”. Upon arrival to the emergency room, the patient was still having severe pain localized at the site of sting.

A physical examination revealed a young male patient who was conscious, oriented but anxious. He looked sick, sweaty, pale and dyspnic. He was hypotensive with a blood pressure of 75 mmHg systolic and 55 mmHg diastolic, and tachycardiac with regular heart beats of 125 beat per minute. He had raised jugular venous pressure, bilateral diffuse lung fields crepitations up to the mid-zones, normal first and second heart sounds and summation gallop. No focal neurological deficits.

By this time patient had received the scorpion anti-venom (1 vial intravenous), IV hydrocortisone 200 mg, and 2000 ml of normal saline over 4 hrs. The IV fluids were stopped when the patient developed signs of pulmonary edema.

**Figure 2.**
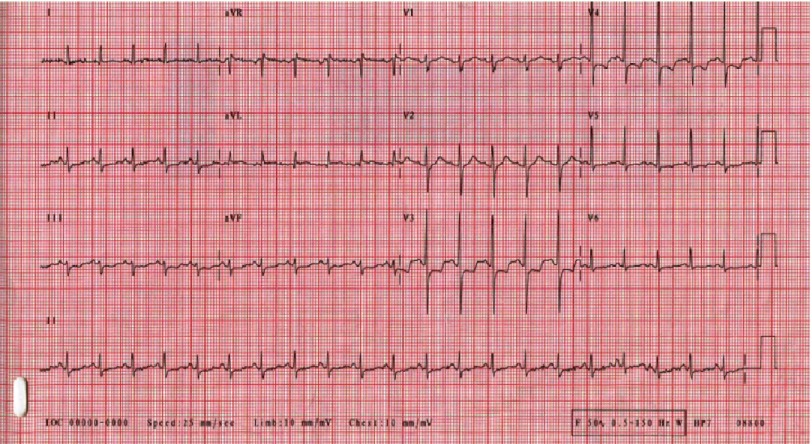
Electrocardiography (ECG) revealing sinus tachycardia with diffuse repolarization abnormalities.

**Figure 3.**
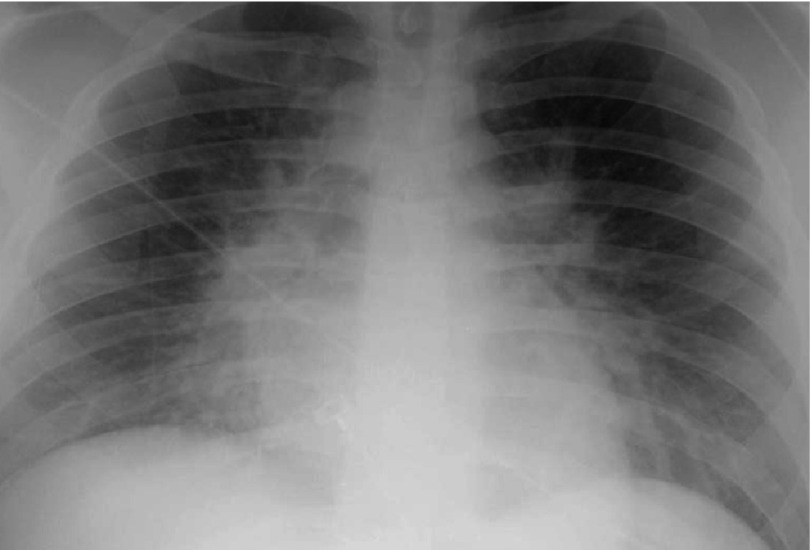
Chest x-ray showing moderate to severe pulmonary edema with borderline heart size.

ECG showed sinus tachycardia with diffuse ST segment depression (Figure 2). Cardiac markers came positive (Troponin T: 0.26–1.3 ng/ml, CK-MB: 52 ng/ml) and pro BNP was elevated (4216 pg/ml). Chest radiography showed moderately severe lung congestion (Figure 3). Urgent echocardiography revealed moderate to severe left ventricular global hypokinesia and impaired systolic function (ejection fraction of 35%) with mild mitral regurgitation and normal study otherwise. Patient was admitted to CCU with an admission diagnosis of toxic myocarditis and cardiogenic shock.

**Table 1.** Major scorpion offenders.

USA: *Centroides exilicauda*
Brazil, S. America: *Tityus serrulatus*
Mexico: *Centroides sufusus*
India: *Buthus tamulus*
Spain: *Buthus occitanus*
Saudia Arabia: *Leiurus quinquestriatus, Androctonus crassicauda*
Middle East: *Leiurus quinquestriatus, Buthus minax, Androctonus*
N. africa: *Androctonus australis, Buthus occitanus, Leuirus*
S. Africa: *Androctonus crassicauda*
Persian Gulf: *Androctonus crassicauda*

**Figure 4.**
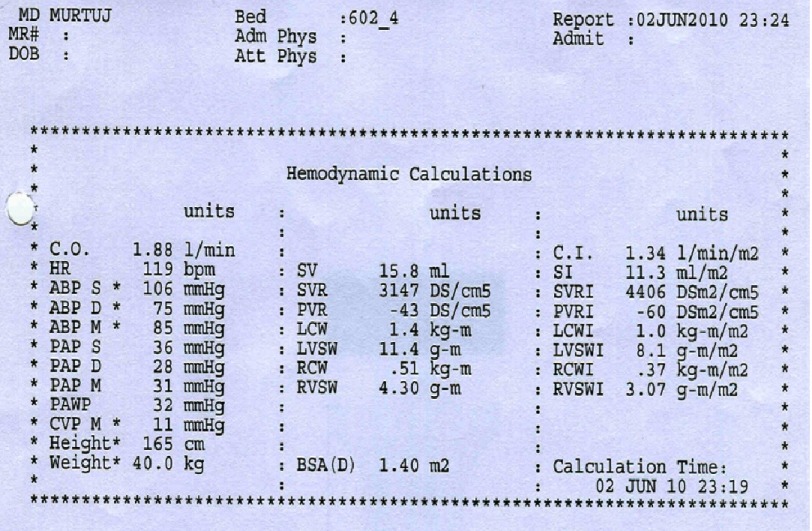
Hemodynamic parameters suggestive of cardiogenic shock.

The patient was intubated, mechanically ventilated and maintained on titratable doses of both inotropic agents noradrenaline and dopamine, and maintenance doses of IV hydrocortisone and antibiotics (piperacillin plus tazobactam). Right heart catheterization suggested a picture of cardiogenic shock with cardiac output of 1.88 l/min (normal: 4.5–7 l/min), cardiac index of 1.34 l/min/m^2^ (normal: 2.5–4.2 l/min/m^2^), systemic vascular resistence of 3150 d s/cm^5^ (normal: 800–1200 d s/cm^5^), and pulmonary capillary wedge pressure of 32 mmHg (normal: 6–12 mmHg) (Figure 4).

**Figure 5.**
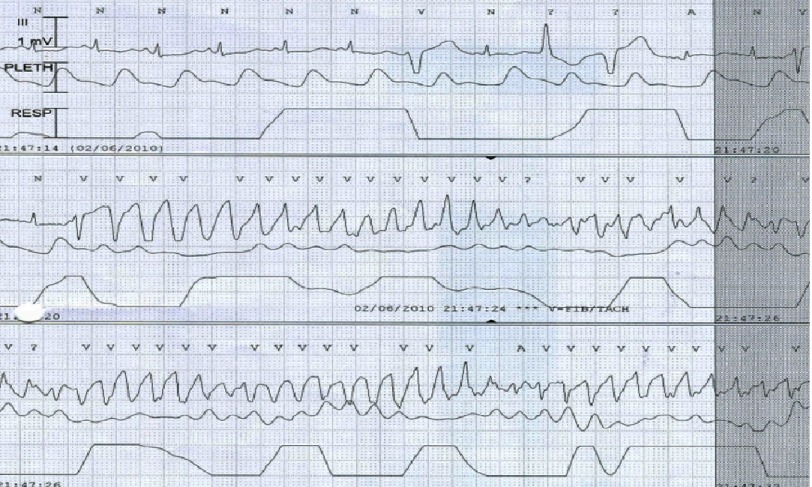
ECG monitor tracing showing short run of polymorphic V-Tac.

**Figure 6.**
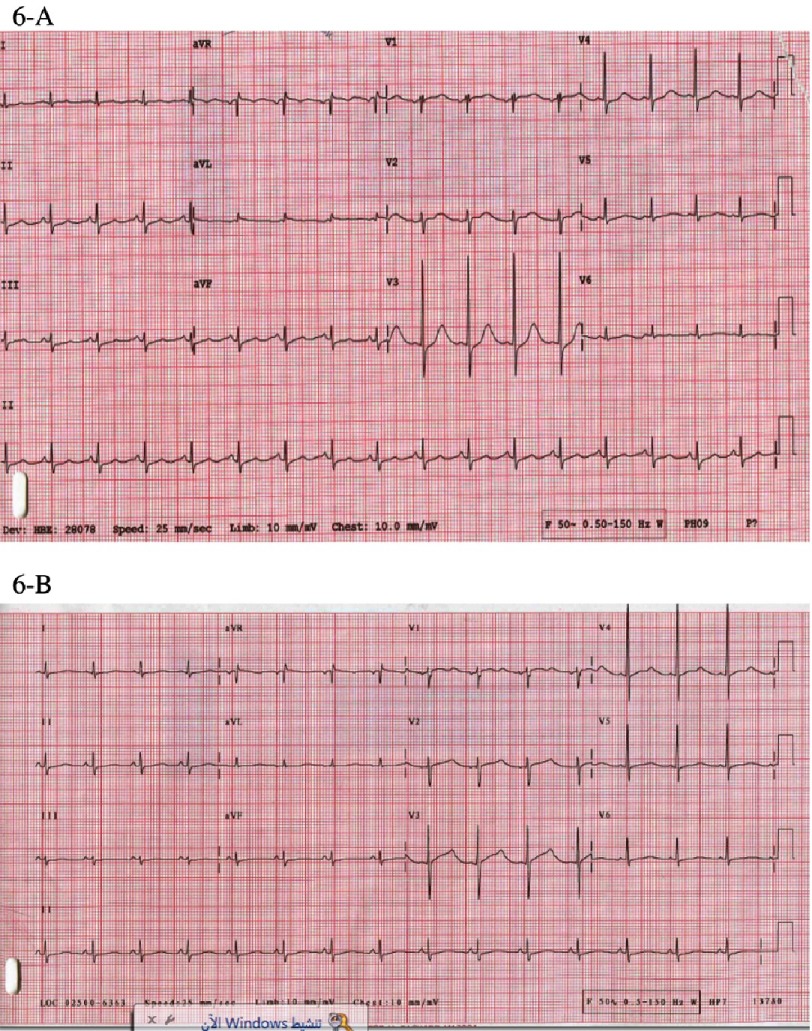
(A) ECG 2 days after admission, revealing sinus tachycardia with resolution of the initial ST-T changes and a prolonged Q-T interval. Corrected Q-T (QTc = 503 ms). (B) Repeated ECG 5 days after admission showing partial normalization of Q-T interval (QTc = 471 ms).

**Figure 7.**
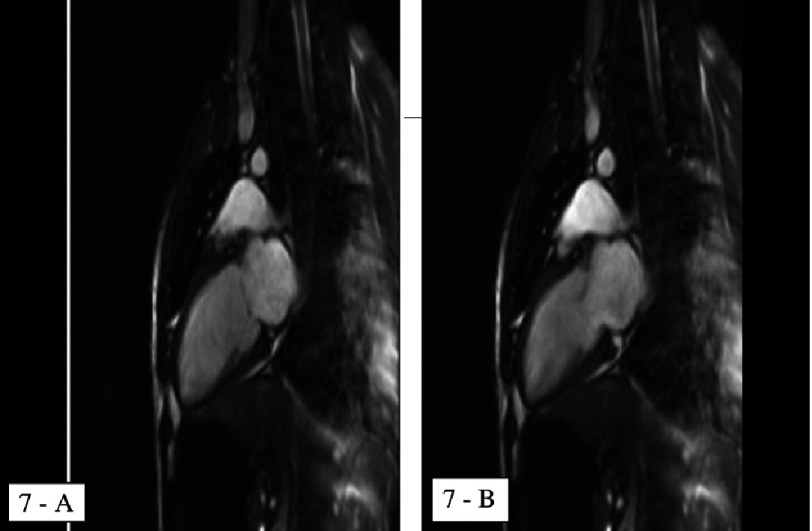
Cardiac MRI showing global LV hypokinesia. 7a: 2-chamber view end-diastolic, 7b: 2-chamber view end-systolic.

**Figure 8.**
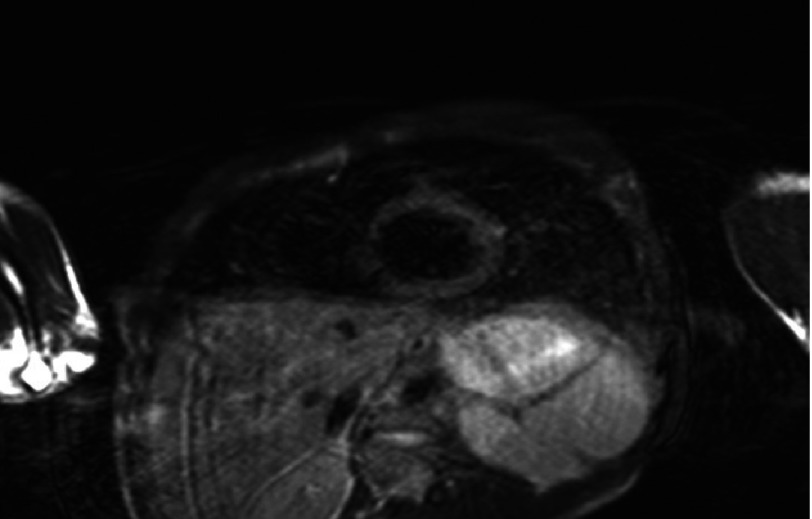
Cardiac MRI (Dark Blood T2WI) shows diffuse high signal intensity within the myocardium (arrow) representing a diffuse myocardial edema (acute myocarditis).

Next day, the patient was febrile with a temprature of 39°C. He developed short runs of ventricular tachycardia (Figure 5) which spontaneously resolved. Two days after admission, his ECG showed sinus rhythm with resolution of the ST segment changes and prolongation of QT interval, corrected QT interval was 503 ms (Figure 6a), while the QT interval was partially normalized five days after admission with a corrected QT interval of 471 ms (Figure 6b). Prazocin (1mg orally twice daily) was then started after hemodynamic stabilization and withdrawal of inotropic agents. Cardiac MRI with gadolinium contrast was arranged, and revealed global LV hypokinesia and systolic dysfunction with global myocardial edema suggestive of diffuse myocarditis; no evidence of myocardial scar by delayed gadolinum enhancement views (Figures 7 and 8).

After one week of CCU course, patient was off-sedation, fully conscious, afebrile wih stable hemodynamics. He was maintaining good oxygen saturation on 40% FiO_2_, with clear lung fields. He was then successfully extubated.

**Figure 9.**
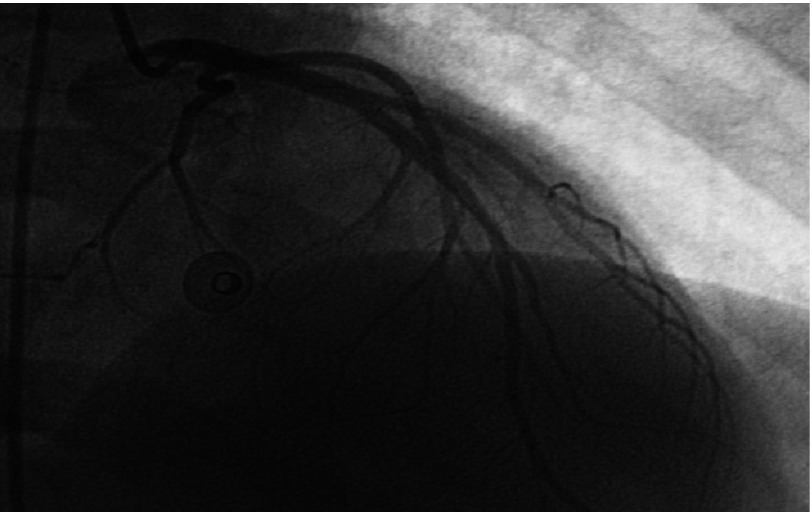
Left coronary artery angiography shows normal vessels.

**Figure 10.**
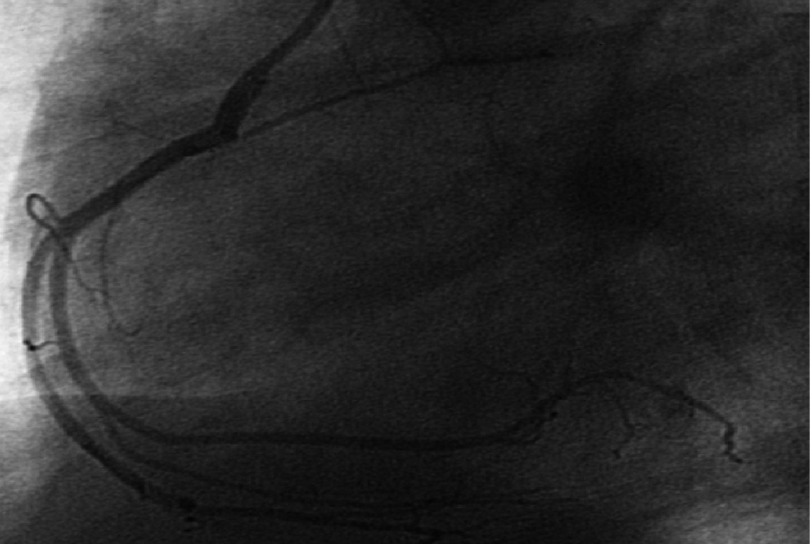
Right coronary artery angiography showing normal study.

Two days later, echocardiography revealed significant improvement with normal LV systolic function (EF: 55%). Coronary angiography was normal for both left and right coronary arteries (Figures 9 and 10). Patient was discharged home in a stable condition, on proton pump inhibitor (Rabiprazole 20mg once/day) and a plan for outpatient follow up.

**Table 2.** Summary of remarkable reviews, their countries, number of patients, purpose of review, and results.

	Country	No of Pts	Purpose	Results
Karnad DR	India	8	Hemodynamic profiles	1 hypertensive/tachycardia
				7 hypotensive/pulmonary edema/cardiogenic shock
				1 mortality
Sofer S, et al.	Israel	32 (children)	Myocardial injury	13 pt (40%) myocardial damage (high enzymes)
				6 pt (18-20%) ECG abnormalities
Mazzei de Dàvila CA, et al.	Venzuella	16 (with LV dysfuction)	Relation between LV dysfunction & Sympathetic activity	All cases with some degree of LV dysfunction have high levels of NE.
Abroug F, et al.	Tunisia	9 (with CHF)	Presentation & Outcomes	All with LV dysfunction (EF: 26 ± 12 %)
				All with CHF (PCWP: 24 ± 2 mmHg).
				8 improved/full recovery
				1 mortality
Ismail M, et al.	Saudi Arabia	3800 (all scorpion sting cases)	Antivenome administration & Outcome	The earlier the Anti-venome given the better the outcome.
				Nigligible CVS manifestations
Bawaskar HS, et al.	West india	658	Prazocin & Outcome	>20% mortality in pre-Prazocin era
				<1% mortality after Prazocin

**Notes.**

* Pt = patient / ECG = Electrocardiography / LV = Left Ventricle / NE = Norepinephrin / CHF = Congestive Heart Failure / EF = Ejection Fraction / PCWP = Pulmonary Capillary Wedge Pressure / CVS = Cardio Vascular System.

## Discussion

Clinical manifestation of scorpion envenomation varies from a localized pain in the site of the sting to a severe generalized intoxication. Cardiovascular involvement include hemodynamic disturbances with hypertensive phase and tachycardia dominating the majority of cases (including *Leiurus Quinquestriatus* envenomation), this can be associated with hypertensive encephalopathy. Occasionally hypotension with or without bradycardia is the dominating hemodynamic picture^1,2^.

In a review of hemodynamic patterns in eight patients in India, vascular constriction with marked hypertention was prominent in one case, thought to have mild envenomation. In the other seven cases, with potentially more severe envenomation, the LV systolic dysfunction with pulmonary edema was the dominating picture with marked tachycardia and variable degrees of hypotention, preceded in some cases by a brief hypertensive phase. One fatal cardiogenic shock was reported^2^.

Pulmonary edema in another review ranged from 7% to 46%, with cardiogenic shock observed in a large number of these cases. Cardiogenic shock usually follows, and occasionally precedes, the hypertensive phase. Cardiac arrest was observed in around 7% of cases^3^.

Sinus tachycardia, variable ST-T changes and prolonged QTc are the most common ECG changes reported in scorpion envenomation. Other changes include bradycardia, myocardial infarction-like changes, conduction abnormalities and rhythm disturbances^1^. Life-threatening ventricular arrhythmias were reported as well^4^. Laboratory findings include elevated cardiac markers (CK, CK-MB, Troponin, SGOT), and elevated plasma and urine chatecolamines. In one review of 32 children following *Leiurus Quinquestriatus* envenomation, 13 out of 32 patients (40.6%) had evidence of myocardial damage manifested by elevated cardiac enzymes (CK, CK-MB, SGOT) and CK-MB/CK ratio > 6%, while only 6 out of these 13 patients had ECG changes suggestive of myocardial injury^5^.

Both echocardiography and nuclear scintigraphy show global hypokinesia with LV systolic and diastolic dysfunction in the majority of cases, most of which were found to improve gradually till complete resolution^1^. In a 16 patient review in Venzuella, this echocardiographic finding of diffuse hypokinesia was associated with higher plasma levels of noradrenaline. With gradual clinical improvement, both noradrenaline levels and LV contractility with systolic function were normalized simultaneously^6^. A cohort study of nine patients with scorpion (*Androctonus australis*) envenomation in Tunisia found a correlation between the depressed LV systolic function, which was reported in all 9 patients (EF: 26 +∕ − 12%), and the hemodynamic profile which was in all patients suggestive of acute congestive heart failure (PCWP: 24 +∕ − 2 mmHg). Other echocardiographic findings included transient mitral regurgitation and LV diastolic function. One mortality case was reported, otherwise the other 8 patients improved gradually over 5-7 days untill complete recovery^7^.

In our case, which is the first to reported in Qatar, the initial presentation was hypotention, tachycardia and heart failure. However there were a few hours prior to the patient’s presentation during which his hemodynamic picture was unknown.

The invasive hemodynamic study completed immediately after admission confirmed the cardiogenic rather than anaphylactic or septic etiology of his shock status. Laboratory, electrocardiographic, and echocardiograhic findings were compatible with the commonly reported findings in previous similar cases.

Cardiac MRI, which was not one of the routine investigations for previously reported cases, was done for our case and revealed global LV hypokinesia and impaired systolic function with diffuse myocardial edema suggestive of acute myocarditis (Figures 6 and 7). The late gadolinium enhancement study did not show any evidence of myocardial scars.

The markedly elevated cardiac markers as well as the significant ST segment changes in the ECG made ischemic etiology (acute coronary syndrome) a possibility, however the echocardiographic and cardiac MRI findings, i.e. the absence of both segmental wall motion abnormalities and myocardial scarring were against this possibility, putting the inflammatory (toxic) etiology on top of deferrential diagnosis list. The normal coronary angiography proved this result later on.

As in the majority of reported cases, the gradual and full improvement and resolution of the clinical, hemodynamic, electrocardiographic and echocardiographic abnormalities was observed in our case.

Management of scorpion envenomation consists of supportive measures and close monitoring, anti-venom adminstration, and Prazocin. Supportive measures include admission to intensive care unit, close monitoring, good hydration and fluid balance, mechanical ventilation and respiratory support, cautious use of inotropic agents if needed, steroids and antibiotics as needed. Inotropic agents role is questionable since their effect of hemodynamic support has no proved mortality benefit^8^.

Early administration of anti-venom has been associated with both less cardiovascular manifestations and lower plasme noradrenalin levels^6^. In a review of the Saudi Arabian experience with antivenom (serotherapy) which involved more than 3,800 cases of scorpion stings, it was established that the incidence of severe venom toxicity was almost negligible in the patients who received early antivenom with reduced hospital stay duration. No significant side effect or serious reactions were reported from the serotherapy in this Saudi experience^9^.

Prazocin, an alpha adrenergic blocker, was found to add a significant benifit to the outcomes of scorpion sting management. In a review from west India, the mortality rates in the pre-prazocin era (1961-1983) were ranging from 25% to 30%. Later on with prazocin therapy the mortality rate was reduced significantly to less than 1%. Prazocin was then considered as an antidote to scorpion venom^8,10^. Similar encouraging results were reported in Saudi Arabia for patients who received prazocin after sub-optimal outcomes with antivenom therapy^11^.

Table 2 summarizes the mentioned reviews regarding their countries, number of patients, purpose of review and remarkable results.

## Conclusion

Myocaritis, pulmonary edema, and cardiologic shock are reported complications of scorpion envenomation. Supportive measures, early anti-venom administration and Prazocin therapy are the main management lines. Cardiac MRI is recommended, when available, as one of the main diagnostic tests in such cases.
